# Yttrium Oxide nanoparticles induce cytotoxicity, genotoxicity, apoptosis, and ferroptosis in the human triple-negative breast cancer MDA-MB-231 cells

**DOI:** 10.1186/s12885-023-11649-w

**Published:** 2023-11-27

**Authors:** Basant Emad, Amr Ahmed WalyEldeen, Hebatallah Hassan, Marwa Sharaky, Ismail A Abdelhamid, Sherif Abdelaziz Ibrahim, Hanan RH Mohamed

**Affiliations:** 1 Biotechnology/Biomolecular Chemistry program, Chemistry Department, Faculty of Science, Cairo University, Giza, 12613 Egypt; 2 Department of Zoology, Faculty of Science, Cairo University, Giza, 12613 Egypt; 3 Pharmacology Unit, Cancer Biology Department, National Cancer Institute, Cairo University, Cairo, 11796 Egypt; 4 Chemistry Department, Faculty of Science, Cairo University, Giza, 12613 Egypt

**Keywords:** Yttrium oxide nanoparticles, Apoptosis, ROS, DNA damage, TNBC, MDA-MB-231

## Abstract

**Background:**

Triple-negative breast cancer (TNBC) is a lethal mammary carcinoma subtype that affects females and is associated with a worse prognosis. Chemotherapy is the only conventional therapy available for patients with TNBC due to the lack of therapeutic targets. Yttrium oxide (Y_2_O_3_) is a rare earth metal oxide, whose nanoparticle (NPs) formulations are used in various applications, including biological imaging, the material sciences, and the chemical synthesis of inorganic chemicals. However, the biological activity of Y_2_O_3_-NPs against TNBC cells has not been fully explored. The current study was conducted to assess Y_2_O_3_-NPs’ anticancer activity against the human TNBC MDA-MB-231 cell line.

**Methods:**

Transmission electron microscopy (TEM), X-ray diffraction, Zeta potential, and dynamic light scattering (DLS) were used to characterize the Y_2_O_3_-NPs. SRB cell viability, reactive oxygen species (ROS) measurement, single-cell gel electrophoresis (comet assay), qPCR, flow cytometry, and Western blot were employed to assess the anticancer activity of the Y_2_O_3_-NPs.

**Results:**

Our results indicate favorable physiochemical properties of Y_2_O_3_-NPs (with approximately average size 14 nm, Zeta Potential about − 53.2 mV, and polydispersity index = 0.630). Y_2_O_3_-NPs showed a potent cytotoxic effect against MDA-MB-231 cells, with IC50 values of 74.4 µg/mL, without cytotoxic effect on the normal retina REP1 and human dermal fibroblast HDF cell lines. Further, treatment of MDA-MB-231 cells with IC50 Y_2_O_3_-NPs resulted in increased oxidative stress, accumulation of intracellular ROS levels, and induced DNA damage assessed by Comet assay. Upon Y_2_O_3_-NPs treatment, a significant increase in the early and late phases of apoptosis was revealed in MDA-MB-231 cells. qPCR results showed that Y_2_O_3_-NPs significantly upregulated the pro-apoptotic genes *CASP3* and *CASP8* as well as ferroptosis-related gene heme oxygenase-1 (*HO-1*), whereas the anti-apoptotic gene *BCL2* was significantly downregulated.

**Conclusion:**

This study suggests that Y_2_O_3_-NPs are safe on normal REP1 and HDF cells and exhibited a potent selective cytotoxic effect against the TNBC MDA-MB-231 cells through increasing levels of ROS generation with subsequent DNA damage, and induction of apoptosis and ferroptosis.

**Supplementary Information:**

The online version contains supplementary material available at 10.1186/s12885-023-11649-w.

## Introduction

In 2020, breast cancer was the major cause of cancer incidence worldwide, with an estimated 2.3 million new cases, representing 11.7% of all cancer cases. Breast cancer accounts for 1 in 4 cancer incidences in women [1]. Also, with 685,000 fatalities, breast cancer is the top cause of cancer-related mortality in women globally, accounting for 1 in 6 cancer-related deaths [1]. There are four intrinsic subtypes of breast cancer: luminal A (estrogen receptor ER+, progesterone receptor PR+, and human epidermal growth factor receptor 2 HER2), with a KI67 index of less than 14%; luminal B has a KI67% of more than 14% (ER+, PR+, HER2- or HER2+); HER2-enriched (ER-, PR-, and HER2 overexpression); and basal-like or triple-negative breast cancer (TNBC), which lacks the three hormonal receptors (ER-, PR-, and HER2-) [2–4]. TNBC patients had greater early recurrence rates, worse disease-specific survival, a more aggressive progression, and more visceral and central nervous system metastases [5]. Although there are no therapeutic options for TNBC patients, the discovery of novel therapeutic agents and the development of targeted therapy provide hope for breast cancer patients in the future.

In cancer therapy, nanotechnology has been intensively investigated and used. Since nanoparticles play an important role as a drug delivery mechanism compared to traditional chemotherapy, nanoparticle-based drug delivery offers distinct benefits, such as better stability and biocompatibility, greater permeability, retention effect, and accurate targeting [6].

Metal oxide nanoparticles exhibit unique applications owing to their acceptable physiochemical features. Yttrium oxide nanoparticles (Y_2_O_3_-NPs) are rare earth nanomaterials that have gained much attention due to their exceptional properties. These properties include a high refractive index, a large band gap (5.8 eV), a high dielectric constant, and high thermal stability [7, 8]. Because of the observed properties and effectiveness, Y_2_O_3_ offers promising use in biomedicine. According to the findings of Nagajyothi et al., Y_2_O_3_ had anticancer properties when tested against cell lines of kidney cancer cells Caki-2 [9].

Accordingly, Y_2_O_3_-NPs are gaining interest as anticancer and in biomedicine candidates. Therefore, the present study was undertaken to test the cytotoxic and genotoxic activities of Y_2_O_3_-NPs against the human TNBC MDA-MB-231 and normal cells employing different experimental approaches.

## Materials and methods

### Chemicals

Y_2_O_3_-NPs were purchased from Sigma-Aldrich (Cat# 205,168, Saint Louis, USA) were suspended in phosphate-buffered saline (PBS) and super-sonicated to prepare the required concentrations for experimentations of this study. All supplies were from sigma, unless otherwise stated.

### Characterization of Y_2_O_3_ NPs

The physicochemical properties of Y_2_O_3_-NPs were determined using a transmission electron microscope (TEM), X-ray diffraction, and dynamic light scattering (DLS) as previously described (10–11).

### Cell culture

The human TNBC cell line MDA-MB-231, normal retina RPE1 (Cell culture core facility, national research center, Dokki-Cairo, Egypt), human dermal fibroblasts (HDF; National cancer institute, Cairo University, Cairo, Egypt) were maintained in DMEM (for MDA-MB-231 and HDF cells) or DMEM-F12 (for RPE1 cells) media supplemented with 1% antibiotic-antimycotic (penicillin and streptomycin) mixture, 1% L-glutamine, and 10% fetal bovine serum (FBS). Cells were maintained at 37 °C in a humidified incubator with 7.5% CO_2_ for MDA-MB-231 and 5% for HDF and REP1 cells.

### Determination of cell viability using sulphorhodamine B (SRB) colorimetric assay

MDA-MB-231, REP1, and HDF cells were seeded at a concentration of 4–10 × 10^3^ cells/well in fresh complete growth medium in 96-well microtiter plastic plates at 37º C for 24 h as previously described [12, 13]. The media were then aspirated, fresh medium (without serum) was added, and cells were incubated either with PBS as vehicle (negative control) or with Y_2_O_3_-NPs at serial different concentrations of 100, 50, 25, 12.5, 6.25, 3.125, 0.78, and 1.56 µg/mL. After 48 h of incubation, the cells were fixed with 10% trichloroacetic acid (TCA) and kept at 4 °C for 1 h. After washing with distilled water, the fixed cells were stained with 0.4% SRB dissolved in 1% acetic acid for 30 min in dark. Unbound dye was removed by washing with 1% acetic acid and plates were left to dry. Bound dye was dissolved in 10 mM Tris-base (pH 10.5) and optical density was read spectrophotometrically at 570 nm using an ELISA microplate reader (Sunrise Tecan reader, Germany). IC50 was determined using GraphPad software (version 8) in three replicates.

### Treatment schedule

Cancerous MDA-MB-231 and normal REP1 and HDF cells were seeded in a T25 flask at a density of 4 × 10^5^, and on the next day the flasks were treated with Y_2_O_3_-NPs at a concentration of 74.4 µg/ mL. The flasks were incubated at 37° C and CO_2_ (7.5% for MDA-MB-231 and 5% for HDF and REP1 cells) for 48 h. After incubation, the media was removed and flasks were washed with PBS and trypsin was added to detach the cells. Trypsin was then inactivated and cells were centrifuged at 200 ×g for 5 min to have pellets. Three replicates were done for each treatment. Pellets were washed once by PBS and preserved in PBS at -80 ºC for further molecular studies.

### Alkaline single-cell gel electrophoresis (comet assay)

DNA damage was assessed using alkaline comet assay and performed analogously to our previous study [14]. The treated and untreated cancer MDA-MB-231 and normal REP1 cells were suspended in PBS solution. Cell suspension (15 µL) of each sample, mixed with (80 µL) of 1% low melting agarose, spread uniformly on a slide fully frosted and coated with normal melting point agarose (1%). Eventually, the slides were imaged and analyzed using COMETSCORE software.

### Apoptosis assay

Flow cytometry was used to assess apoptosis using Fluorescein isothiocyanate (FITC)-Annexin V Apoptosis detection Kit (BD Biosciences, California; United States). Control and IC50 Y_2_O_3_-NPs-treated cells were collected and washed two times with cold PBS and then re-suspended in 1X binding buffer. Subsequently, 5 µL FITC Annexin V and 5 µL of Propidium Iodide (PI) were added and the cells were then incubated at RT in dark for 15 min. Finally, 400 µL of Binding buffer was added and 50,000 events were acquired immediately using Cytoflex (Beckman coulter, USA). Data were analyzed using FCS express 7 software (De Novo software, Pasadena, CA, USA).

### Quantitative real-time PCR

Total RNA was isolated using The GeneJET RNA Purification Kit (Thermo Fisher Scientific, USA) from cultured cells, and cDNA was synthesized using 1 µg RNA by the cDNA Reverse Transcription Kit (Applied Biosystems, Foster City, CA, USA). Specific gene expression was quantified using SYBR^TM^ Green PCR Master Mix (Applied Biosystems, USA) in StepOnePlus Real-Time PCR System (Applied Biosystems). The relative expression of genes was determined using the formula for fold change 2^−ΔΔCT^. Primer sequences are shown in Table 1.

**Table 1:** The primer sequences used in quantitative real-time PCR

Gene		Primer’s sequences
*β-ACTB*	F	TCCCTGGAGAAGAGCTACG
	R	GTAGTTTCGTGGATGCCACA
*BCL-2*	F	TCCGATCAGGAAGGCTAGAGT
	R	TCGGTCTCCTAAAAGCAGGC
*CASP8*	F	GAT CAA GCC CCA CGA TGAC
	R	CCT GTC CAT CAG TGC CATAG
*CASP3*	F	GGAAGCGAATCAATGGACTCTGG
	R	GCATCGACATCTGTACCAGACC
*p53*	F	CCTCAGCATCTTATCCGAGTGG
	R	TGGATGGTGGTACAGTCAGAGC
*LC3B*	F	GAGAAGCAGCTTCCTGTTCTGG
	R	GTGTCCGTTCACCAACAGGAAG
*TF*	F	TCACTCCTGGAAGCCTGCAC
	R	CACTTGGGCCAGTGAAACCA
*HO-1*	F	GGGTGATAGAAGAGGCCAAGA
	R	AGCTCCTGCAACTCCTCAAA

### Western blot

Total cell lysates were prepared in RIPA buffer containing protease inhibitor cocktail. Protein concentration was determined by BCA assay and equal protein concentrations were then separated using 15% SDS-PAGE. After proteins transfer, a nitrocellulose membrane (Amersham GE healthcare life sciences, USA) was blocked with 5% skimmed milk in TBST for 1 h and washed three times. The membrane was cut and one part was incubated with primary antibody Survivin (SC-17779, Santa Cruz, TX, USA) and the other part was probed with antibody against β-actin as a loading control (SC-47778, Santa Cruz) at dilution 1:1000 overnight at 4 °C. Following washing three times, the membrane was incubated with horseradish peroxidase (HRP)-conjugated anti-mouse secondary antibody (Sigma Aldrich) for 1 h. Chemiluminescent signals were developed with ECL (Thermo Scientific, Waltham, USA) using UVP Biospectrum Imaging System (Analytik Jena, Cambridge, UK). Band intensities were quantified using ImageJ software (National Institutes of Health, Bethesda, MA, USA).

### Measurement of intracellular ROS generation and oxidative stress markers

The level of ROS production within untreated and Y_2_O_3_-NP-treated cancer MDA-MB-231 and normal retina REP1 cells was studied using 2,7-dichlorofluorescin diacetate (DCFH-DA) [15]. Cells were incubated for 30 min in dark with 20 mM 2,7-dichlorofluorescin diacetate (DCFH-DA), which passively enters the cells and reacts with ROS creating the highly fluorescent chemical compound dichlorofluorescein (DCF). Fluorescent cells were then inspected with a fluorescent microscope and photographed at a 200X magnification.

The level of malondialdehyde (MDA), an indicator of lipid peroxidation, was assessed as described before [15, 16]. The end product of the lipid peroxidation process interacts with thiobarbituric acid and results in the formation of pink color complex measured spectrophotometry at 532 nm. The level of antioxidant GSH protein was measured using Elmans method [17]. Elmans reagent was reduced with the -SH group present in GSH producing 2-nitro-s-mercaptobenzoic acid (yellow color) measured spectrophotometrically at 412 nm. The antioxidant CAT enzyme was assessed using Abei method [18]. After allowing a certain amount of hydrogen peroxide to react with CAT for one minute, the process was stopped with a CAT inhibitor. Any hydrogen peroxide remained interacted with 4-aminophenazone and 3,5-dichloro-2-hydroxybenzene sulfonic acid in the presence of peroxidase, producing a chromophore, whose color intensity was inversely correlated with the activity of CAT. The chromophore’s absorbance was measured at 510 nm. All absorbance measurements were performed using a UV–Vis spectrophotometer (U-2001, model 121–0032 Hitachi, Tokyo, Japan).

### Statistical analysis

The results presented for the current study are displayed as mean ± Standard Error of the mean (SEM) and were analyzed using the Statistical Package for the Social Sciences (SPSS) (version 22) at the significance level *P* < 0.05. Unpaired Student’s t-test was used to compare between the untreated and treated cells.

## Results

### Characterization of Y_2_O_3_-NPs

**Fig. 1:**
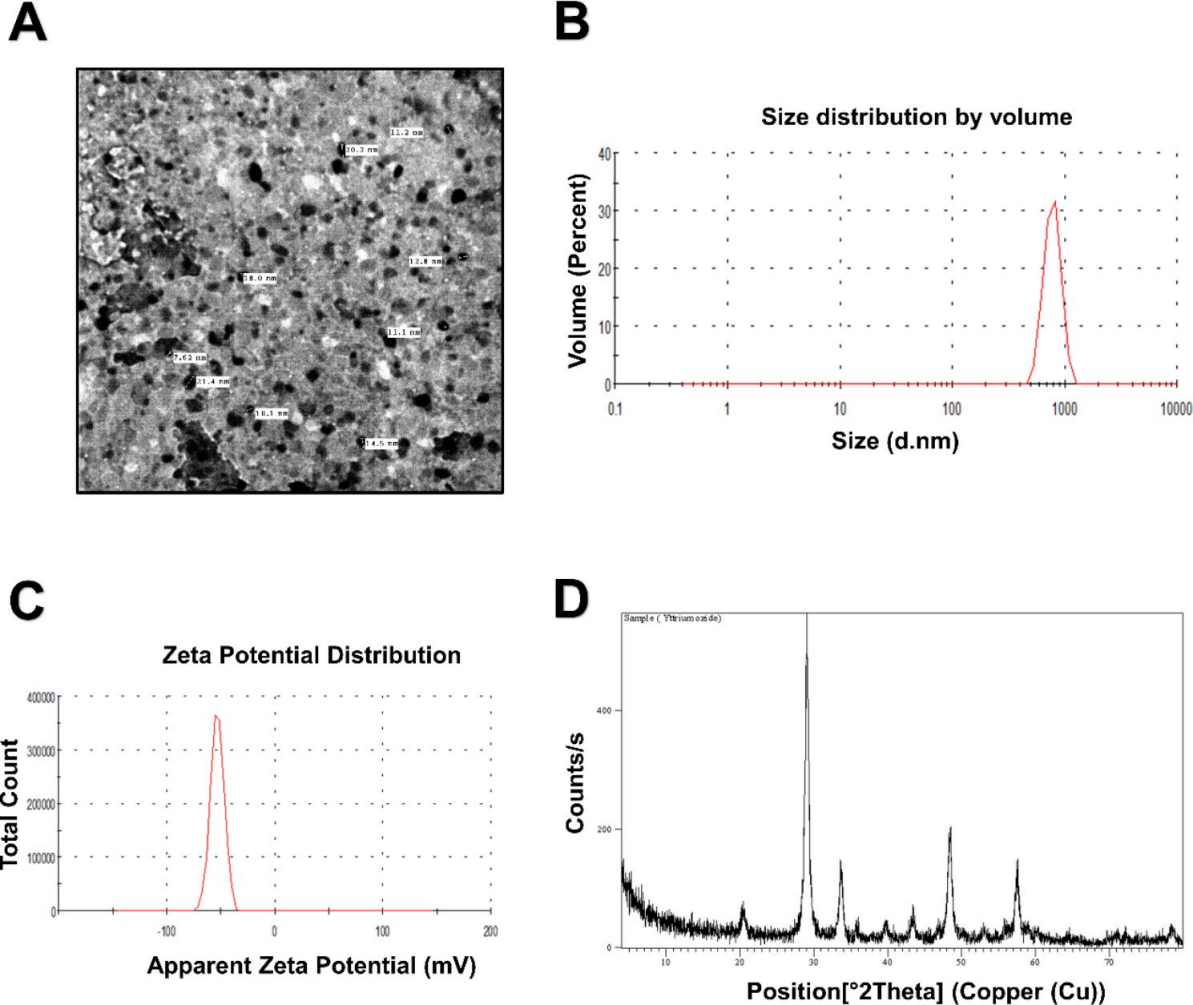
Characterization of Y_2_O_3_-NPs. (**A**) A representative micrograph of Y_2_O_3_-NPs using TEM showing the average size of Y_2_O_3_-NPs = 14 nm. (**B**) Zeta potential of Y_2_O_3_-NPs. **(C)** The hydrodynamic diameter of 100% Y_2_O_3_-NPs using DLS. **(D)** X-ray diffraction of Y_2_O_3_-NPs. Abbreviations: Y_2_O_3_-NPs: Yttrium Oxide nanoparticles. TEM: transmission electron microscope. DLS: Dynamic light scattering

The TEM imaging showed that Y_2_O_3_-NPs are well dispersed and have an average particle size of about 14 nm with a cubic shape (Fig. 1A). The Zeta Potential of Y_2_O_3_-NPs showed a peak at -53.2 mV, which indicates its fairly good stability (Negative charge) (Fig. 1B). The relative percentage of light scattering of the average particle size and the peak was found at 790.6 d.nm and its width 132.9 d.nm, at volume 100%, and temperature 25 °C with a polydispersity index (Pdi)  0.630 (Fig. 1C). The crystal structure of the Y_2_O_3_-NPs was characterized by X-ray diffraction (XRD) and showed a high purity of Y_2_O_3_-NPs with no impurity’s peaks (Fig. 1D).

### Effect of Y_2_O_3_-NPs treatment on cell viability

**Fig. 2:**
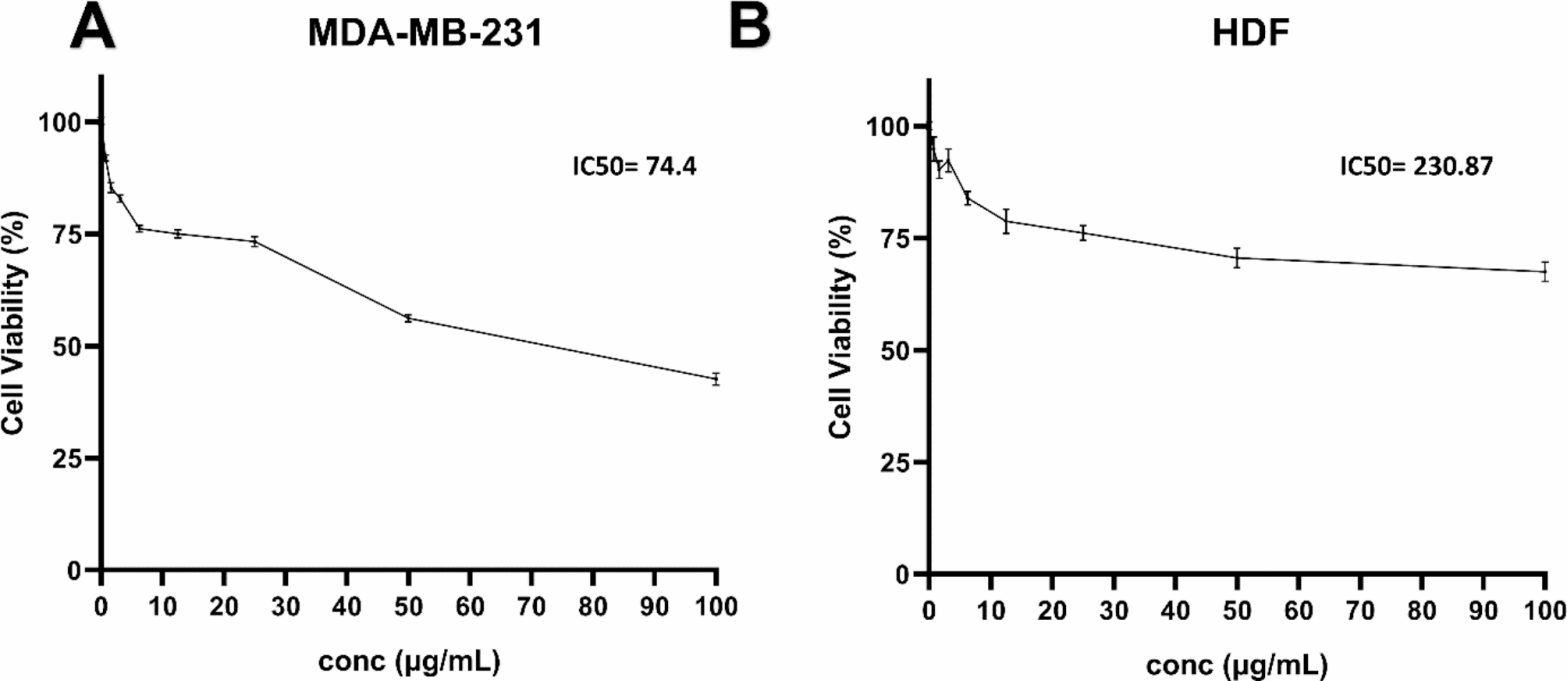
Effect of different concentration of Y_2_O_3_-NPs for 48 h on MDA-MB-231 and HDF cells assessed by SRB assay. (**A**) Effect of Y_2_O_3_-NPs treatment on MDA-MB-231 cell viability. (**B**) Effect of Y_2_O_3_-NPs treatment on HDF cell viability. Data are represented as mean ± SEM and representative of three independent experiments

The SRB assay was used to assess the cytotoxicity of Y_2_O_3_-NPs against the MDA-MB-231, HDF, and RPE1 cells. Y_2_O_3_-NPs showed a selective potent cytotoxic activity against the aggressive TNBC MDA-MB-231 cells with IC50 value of 74.4 µg/mL as depicted in Fig. 2A. Interestingly, Y_2_O_3_-NPs were low/non-toxic against the normal RPE1 and HDF cells with IC50 more than 100 µg/mL (Fig. 2B). Therefore, IC50 74.4 µg/mL Y_2_O_3_-NPs was used for all subsequent experiments.

### Y_2_O_3_-NPs induces DNA damage in MDA-MB-231 cells

**Fig. 3:**
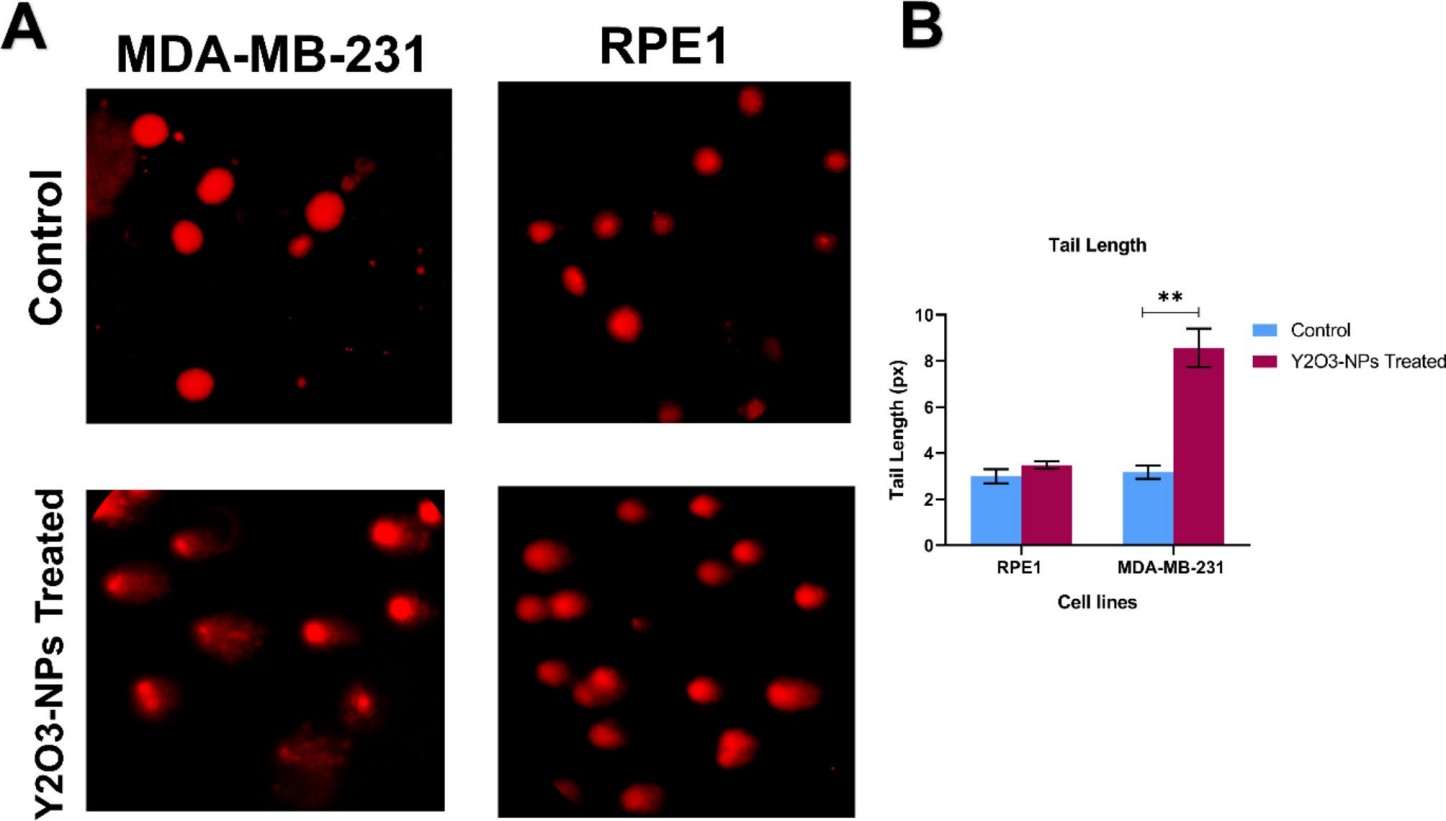
Effect of Y_2_O_3_-NPs on DNA damage in breast cancer MDA-MB-231 cells and normal retina cells REP1. (**A**) Representative images of damaged DNA induced by Y_2_O_3_-NPs treatment compared to untreated control cells. (**B**) DNA damage parameter (tail length (px)). The tail length parameter was measured on ≥ 50 cells. CometScore software (V2.0) was used to assess DNA damage parameters. Bars represent means ± SEM, n = 3. **P < 0.01 as determined via Student’s t-test

The results of alkaline Comet assay demonstrated a selective induction of DNA damage upon treatment with Y_2_O_3_-NPs (74.4 µg/mL) in MDA-MB-231 cancer cells revealed by a significant (*P* < 0.01) increase in tail length compared to untreated MDA-MB-231 cells (Fig. 3). On the contrary, no significant changes were observed in the tail length in normal retina REP1 cells treated with the same concentration of Y_2_O_3_-NPs compared to the untreated retina REP1 cells (Fig. 3).

### Y_2_O_3_-NPs affect oxidative stress and ROS levels in MDA-MB-231 cells

**Fig. 4:**
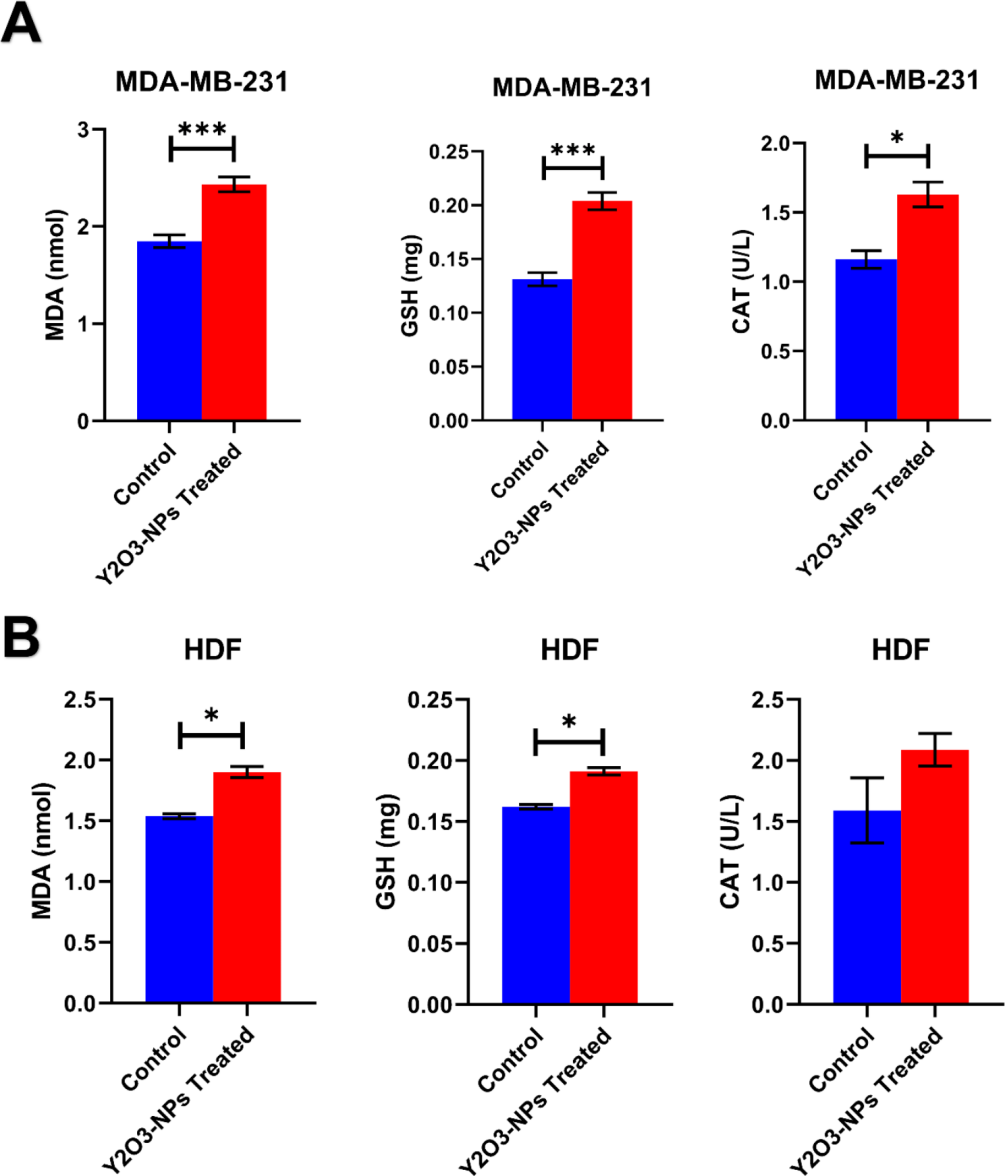
Effect of Y_2_O_3_-NPs on levels of MDA and GSH, as well as the activity of CAT in MDA-MB-231 (**A**) and HDF (**B**) cells. Data represent mean ± SEM, n ≥ 2. * *P* < 0.05, *** *P* < 0.001 as determined by Student’s t-test

Since DNA damage can be resulted from oxidative stress [14], we examined whether Y_2_O_3_-NPs had an influence on ROS production in MDA-MB-231 and REP1 cells using DCFH-DA staining. The results showed a potent effect of Y_2_O_3_-NPs on ROS production in MDA-MB-231 cells. However, the Y_2_O_3_-NPs did not show a similar effect on the normal REP1 cells (Figure S1). Next, we evaluated levels or activity of oxidative stress/ (assessed by MDA) and antioxidant (assessed by GSH and catalase)-related markers upon treatment with Y_2_O_3_-NPs in MDA-MB-231 and normal HDF cells. A significant increase in MDA (by 1.3-fold; *P* < 0.001), GSH (by 1.6-fold; *P* < 0.001), and CAT activity (by 1.4-fold; *P* < 0.01) was observed in Y_2_O_3_-NPs treated MDA-MB-231 cells compared to the untreated MDA-MB-231 cells (Fig. 4A). Similarly, a significant increment in MDA (by 1.2-fold; *P* < 0.05), and GSH (by 1.2-fold; *P* < 0.05) was observed in HDF cells, but CAT activity did not alter upon Y_2_O_3_-NPs treatment (Fig. 4B).

### Y_2_O_3_-NPs induce apoptosis and affect expression of apoptosis- and ferroptosis-related markers in MDA-MB-231 cells

**Fig. 5:**
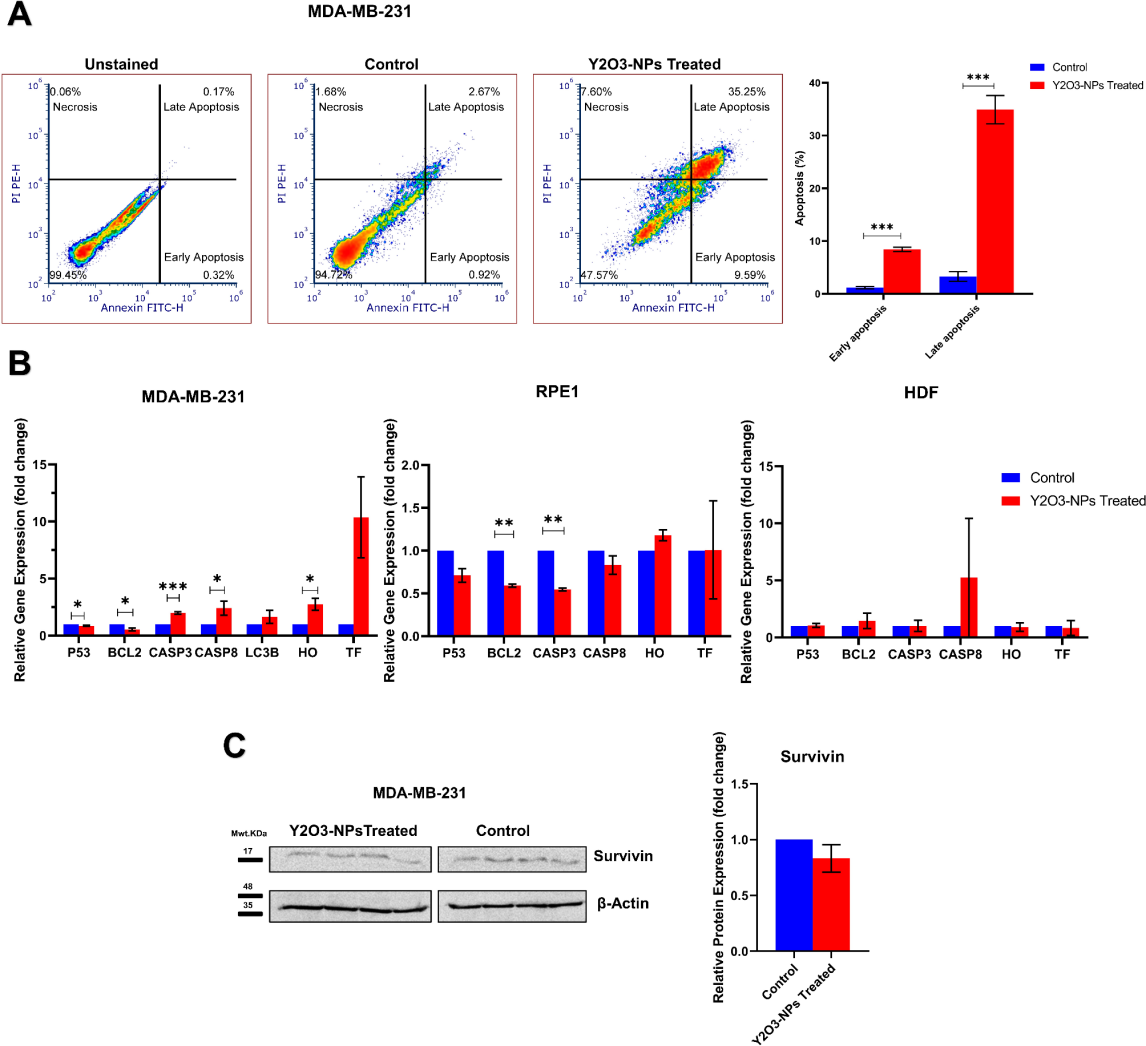
Y_2_O_3_-NPs enhance apoptosis and alter expression of apoptosis- and ferroptosis-related markers. (**A**) A representative flow cytometry-based apoptosis in control and Y_2_O_3_-NPs treated MDA-MB-231 cells (left panel). Quantitative analysis of early and late apoptosis (right panel). Data represent mean ± SEM, n ≥ 2. *** *P* < 0.001 as determined by Student’s t-test. Data shown are a single experiment representative of three independent experiments. (**B**) mRNA expression levels of the pro-apoptotic *CASP3*, *CASP8*, anti-apoptotic *BCL2*, autophagic gene *LC3B*, and ferroptosis-related genes *HO-1 and TF* assessed by qPCR in RPE1, HDF, and MDA-MB-231 cells. Data represent mean ± SEM, n ≥ 3. * *P* < 0.05, ** *P* < 0.01 and *** *P* < 0.01 as determined by Student’s t-test. (**C**) Immunoblotting of Survivin expression in control and Y_2_O_3_-NPs-treated MDA-MB-231 cells (left panel). Following blocking and washing steps, nitrocellulose membrane was cut and probed with the indicated antibodies. Quantification of band intensity (right panel) normalized to β-actin as loading control. Data represent means ± SEM, n = 2

Y_2_O_3_-NPs had an influence on DNA damage, and that may affect apoptosis [14]. Indeed, our flow cytometric analysis uncovered apoptosis induction upon Y_2_O_3_-NPs as manifested by the markedly significant (*P* < 0.001) increase in early and late apoptosis in MDA-MB-231 cells (Fig. 5A).

We further assessed the expression level of key apoptotic, ferroptotic, and antiapoptotic genes in Y_2_O_3_-NPs-treated MDA-MB-231, RPE1 and HDF cells. The qPCR results showed that Y_2_O_3_-NPs treatment in MDA-MB-231 cells significantly upregulated the pro-apoptotic genes, namely *CASP3* (by 1.98-fold; *P* < 0.0001) and *CASP8* (by 2.4-fold; *P* < 0.05), as well as ferroptosis *HO-1* gene (by 2.8-fold; *P* < 0.05), but no significant change in *TF* gene expression was observed. *p53* was down-regulated (by 0.85-fold; *P* < 0.05). Y_2_O_3_-NPs also significantly down-regulated the anti-apoptotic gene BCL2 (by 0.53-fold; *P* < 0.05). In addition, Y_2_O_3_-NPs caused up-regulation in the autophagic gene LC3B (by 1.65-fold); however, it did not reach a significant level (Fig. 5B). On the other hand, gene expression level of *BCL2* (by 0.4-fold; *P* < 0.01) and *CASP3* (by 0.5-fold; *P* < 0.01) was significantly decreased in normal RPE-1 cells upon Y_2_O_3_-NPs treatment (Fig. 5B). We did not observe any changes in the aforementioned gene expression in normal HDF cells (Fig. 5B). We further verified expression of the antiapoptotic survivin on the protein level. Western blot analysis indicated downexpression of survivin in MDA-MB-231 cells treated with Y_2_O_3_-NPs; however, it did not reach the significance level (Fig. 5C).

## Discussion

This study was conducted to evaluate the anticancer activity of Y_2_O_3_-NPs against the TNBC MDA-MB-231 cells and to explore the mechanism of anticancer action of Y_2_O_3_-NPs. In our study, we successfully characterized Y_2_O_3_-NPs by *XRD*, size distribution, Zeta potential, and TEM. Further, we demonstrated the cytotoxic effect of Y_2_O_3_-NPs against the aggressive TNBC MDA-MB-231 cells with no cytotoxicity against normal RPE1 and HDF cells. Consistently, it has been shown that Y_2_O_3_-NPs decrease the viability of the human skin keratinocyte HaCaT [19], human embryonic kidney HEK293 [20], and cultured primary osteoblasts cells [21]. Therefore, our study extends the observation of the cytotoxic effect of Y_2_O_3_-NPs to TNBC MDA-MB-231 cells.

The strong selective cytotoxicity of Y_2_O_3_-NPs demonstrated in this study against MDA-MB-231 cells can be attributed to the elevation of ROS generation in the aggressive MDA-MB-231 cancer cells that disrupting cellular homeostasis and lead to cell death. It has been shown that the reduction of cells viability can be attributed to over generation of intracellular ROS, which are disrupting the balance between oxidants and antioxidants and damaging cellular macromolecules leading to cell death [22, 23]. Indeed, oxidative stress mirrored by the increased MDA levels was detected in MDA-MB-231 treated with Y_2_O_3_-NPs. However, it should be noted that level or activity of the antioxidant markers GSH and CAT was also increased. This may suggest that oxidative stress exerted by Y_2_O_3_-NPs treatment may overrule the antioxidant status.

Overproduction of ROS generation causes severe DNA damage [24]. Our comet assay results showed increased tail length evidenced by increased DNA damage due to elevated oxidative stress and ROS production upon Y_2_O_3_-p53NPs treatment. In agreement, a study reported elevated ROS levels in HaCaT and HEK293 cells upon treatment with Y_2_O_3_-NPs leading to DNA damage [19, 20]. In addition, Yttrium chloride triggered cytotoxicity and a DNA damage response in H9c2 cardiomyocytes via the production of ROS and the suppression of the Nrf2/PPAR pathways [25]. Y_2_O_3_-NPs showed an increase on intercellular ROS production against A375 melanoma cells and eventually caused DNA damage this effect was improved synergistically when combining the Y_2_O_3_-NPs with X-ray irradiation [26].

Excessive DNA damage induces apoptosis [22, 27]. This was further confirmed in our study revealed by increased early and late apoptosis in Y_2_O_3_-NPs-treaed MDA-MB-231 cells relative to untreated control cells. In agreement, our qPCR results showed a significant upregulation of the pro-apoptotic genes *CASP3* and *CASP8* and downregulation of the anti-apoptotic *BCL2* gene. These results are consistent with a study that showed an increase in *CASP3* in osteoblast cells after treatment with Y_2_O_3_-NPs [21]. Likewise, treatment with Y_2_O_3_-NPs increased the ratio of *Bax/Bcl-2* and *CASP3* expression, in a dose and time-dependent manner, and promoted apoptosis in HEK293 cells [20], as well as increased expression level of *CASP3* gene in HaCaT cells [19]. ROS production is linked with induction of ferroptosis [28]. Our qPCR unveiled increased expression of ferroptosis-related *HO-1* MDA-MB-231 treated with Y_2_O_3_-NPs. This is contradicting results of the cytotoxic effects of Y_2_O_3_-NPs, as HO-1 is known as proliferative and anti-apoptotic protein in different cancer models [29]. However, another study demonstrated that HO-1 overexpression in MDA-MB-231 cells, resulting decreased cell proliferation [30], indicative of dual role of HO-1 in cancer and that may be a cancer type context-dependent. Some limitations could be associated with this study including usage only one cell line. However, MDA-MB-231 cells are the most commonly used TNBC cell line with highly aggressive properties [31–33]. Another limitation is the increased oxidative stress MDA marker in normal HDF cells treated with Y_2_O_3_-NPs. However, this could be counterbalanced by the increased antioxidant GSH levels, and that was associated with no obvious effect on DNA damage in normal cells evidenced by the comet assay.

## Conclusion

Overall, our results showed a selective anticancer effect of Y_2_O_3_-NPs against the most aggressive MDA-MB-231 cells via regulation ROS, DNA damage, and expression of apoptosis- and ferroptosis-related genes. Therefore, this study underscores the potential of Y_2_O_3_-NPs as a promising drug against TNBC. Further studies are needed to verify these findings in an animal model.

### Electronic supplementary material

Below is the link to the electronic supplementary material.

Supplementary Material 1

## Data Availability

The datasets used and/or analyzed during the current study are available from the corresponding author on reasonable request.
