# Supplementary material for: Yttrium Oxide nanoparticles induce cytotoxicity, genotoxicity, apoptosis, and ferroptosis in the human triple-negative breast cancer MDA-MB-231 cells
Source: BMC Cancer. 2023 Nov 27;23:1151. doi: 10.1186/s12885-023-11649-w (PMC10680179; doi:10.1186/s12885-023-11649-w)
Supplement: Supplementary file 1 — Supplementary Material 1 [file 12885_2023_11649_MOESM1_ESM.docx]

**Figure S1.** DCFH-DA fluorescence photomicrographs for MDA-MB-231 cells compared with normal REP1 cells upon treatment with Y_2_O_3_-NPs.
